# A Nomogram Predicting the Overall Survival and Cancer-Specific Survival in Patients with Parathyroid Cancer: A Retrospective Study

**DOI:** 10.3389/fendo.2022.850457

**Published:** 2022-05-19

**Authors:** Mei Tao, Shuyan Luo, Xiaoming Wang, Meng Jia, Xiubo Lu

**Affiliations:** ^1^ Department of Thyroid Surgery, The First Affiliated Hospital of Zhengzhou University, Zhengzhou, China; ^2^ Department of Neurology Surgery, The First Hospital of China Medical University, Shenyang, China

**Keywords:** parathyroid carcinoma, prognosis, nomogram, SEER, distant metastasis

## Abstract

**Purpose:**

This study aimed to explore a visual model for predicting the prognosis of patients with parathyroid carcinoma (PC) and analyze related biochemistries in different groups of stage.

**Methods:**

The training dataset of 342 patients with PC was obtained from the Surveillance, Epidemiology, and End Results (SEER) database, and the validation dataset included 59 patients from The First Affiliated Hospital of Zhengzhou University. Univariate and multivariate Cox regression analyses were performed to evaluate significant independent prognostic factors. Based on those factors, nomograms and Web-based probability calculators were constructed to evaluate the overall survival (OS) and the cancer-specific survival (CSS) at 3, 5, and 8 years. The concordance index (C-index), receiver operating characteristic (ROC) curve, calibration curve, and decision curve analysis (DCA) were used to evaluate the nomogram in the training set and validation set. Moreover, biochemistries from the validation set were retrospectively analyzed in different groups of stage by Kruskal–Wallis test.

**Results:**

Age, marital status, tumor size, stage, lymph node status, and radiation were identified as prognostic factors of OS. In contrast, only tumor size and stage were predictive for CSS. The nomogram was developed based on these independent factors. The C-index, ROC curve, calibration curve, and DCA of the nomogram in both training and validation sets showed that the nomogram had good predictive value, stability, and clinical benefit in predicting 3-, 5-, and 8-year OS and CSS in PC patients. Among the 59 PC patients from our hospital, lower albumin (ALB) levels and higher postoperative parathyroid hormone (PTH) levels were found in patients with distant metastasis (Distant vs. Regional ALB levels: *p* = 0.037; Distant vs. Local ALB levels: *p* = 0.046; Distant vs. Regional postoperative PTH levels: *p* = 0.002; Distant vs. Local postoperative PTH: *p* = 0.002).

**Conclusion:**

The established nomogram application can provide accurate prognostics for patients with PC in the Chinese population, but it must be validated on prospectively collected real-world data.

## Introduction

Parathyroid carcinoma (PC) is one of the rarest causes of primary hyperparathyroidism (PHPT), accounting for 0.5%–5% of the total PHPT cases (1). The incidence rate of PC in the population is increasing (2, 3). A meta-analysis reported 234 cases of PC patients in China, and the number of cases increases yearly (4).

Surgery leads to the best chance of survival with a 5-year and 10-year overall survival (OS) rate of 78%–91% and 60%–72%, respectively (1). The cancer-specific survival (CSS) rate was recorded as 89.4%, with a median number of 75 months (3). The indolent course of PC, compared with other invasive tumors, had better survival, but it shows frequent recurrence (1). In a center where the management and patient outcomes of PC have not changed significantly over the past 35 years, the 5-year disease-free survival rate was about 62.3% (5). In addition, almost all patients died from this cancer due to complications of hypercalcemia rather than tumor burden (1, 6). Currently, surgical resection with negative margin therapy is the only effective treatment strategy, as there is no evidence that radiotherapy and chemotherapy are effective for PC (7, 8).

Recently, the 8th American Joint Committee on Cancer (AJCC) staging system adopted Schule’s staging system for tumor patients (9). Another mainstream staging method is proposed by Shaha and Shah (10), including both tumor size and disease progress. Nevertheless, some elements that have been reported to be related to PC, including age, serum calcium levels, intact parathyroid hormone (PTH) levels, vascular invasion, local excision, and absence of parafibromin staining, are not considered in the TNM staging system (2, 11–14). Therefore, to a certain extent, the TNM staging is incomplete and does not provide an individual prediction of PC patients.

Considering the various clinicopathological characteristics that could influence the prognosis of PC, an instrument integrating the relevant predictors is urgently needed for facilitating advanced therapy and improving the quality of life of patients. A nomogram is a simplified visual model for statistical predictions combining independent factors. Many researchers reported possible prognostic factors for PC, but there is no corresponding nomogram.

In this study, prognostic nomograms regarding OS and CSS were developed from PC patient data registered between 2000 and 2018 in the Surveillance, Epidemiology, and End Results (SEER) database. External data from a single Chinese center were utilized to validate these nomograms, and the patient characteristics reported by our hospital were analyzed.

## Methods

### Ethics Statement

This research was approved by the Ethics Committee of the First Affiliated Hospital of Zhengzhou University (Number: 2021-KY-1062-001). Patients from the SEER database had consented to be studied publicly in any scientific research worldwide.

### Population

**Figure 1:**
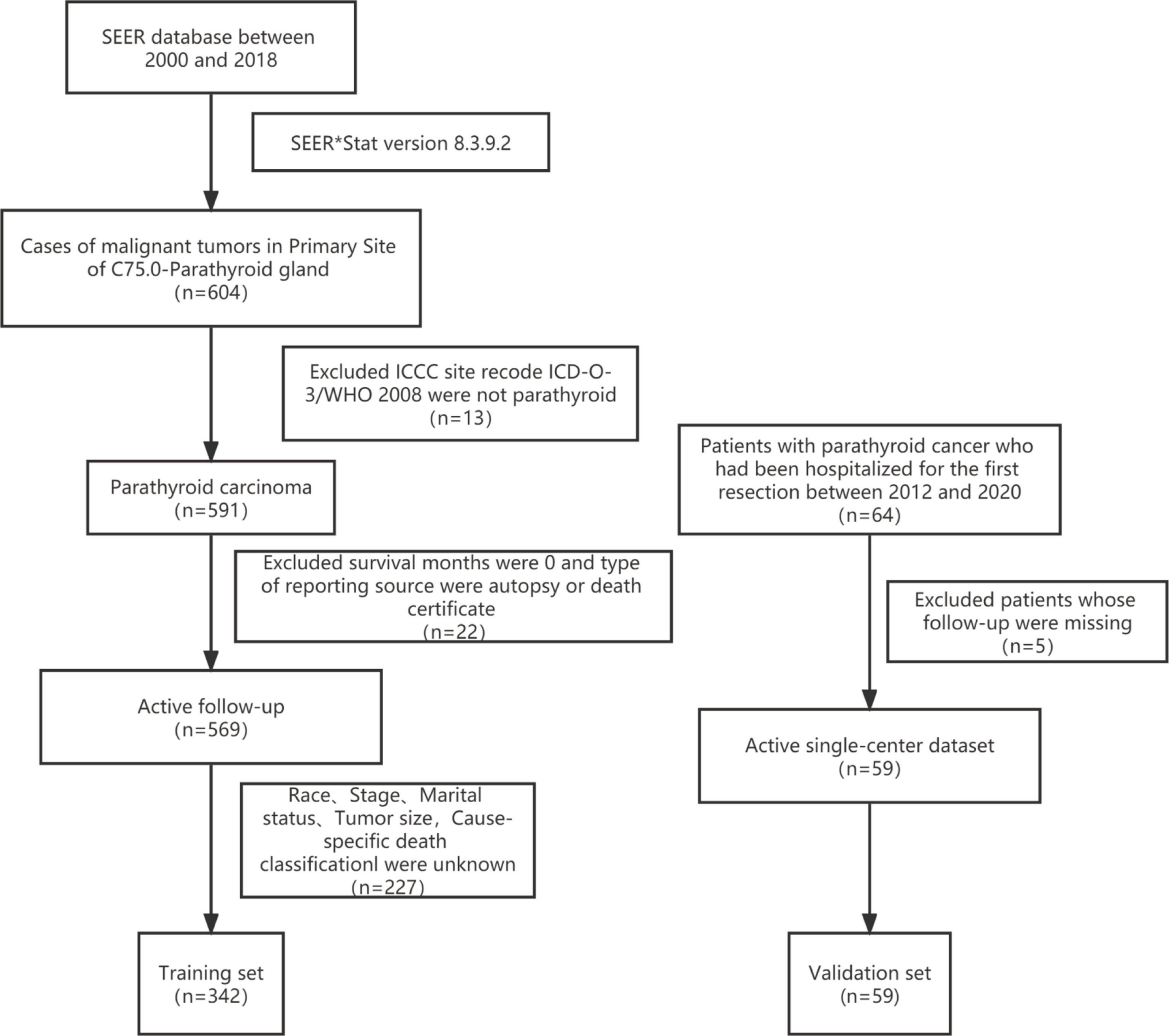
The data process flowchart.

The primary training dataset was downloaded with the SEER*Stat Software (Version 8.3.9.2; National Cancer Institute, Bethesda, MD, USA). The primary site code C75.0 for PC was applied in the SEER research Plus Data,18 Registries, Nov 2020 Sub (2000–2018) database. The exclusion criteria were listed as follows: 1) International Classification of Childhood Cancer (ICCC) site recode International Classification of Disease for Oncology-3 (ICD-O-3)/WHO 2008 was not parathyroid; 2) patients diagnosed at autopsy or *via* death certificates; 3) survival months were 0; 4) unknown race record, stage, surgery, marital status, tumor size, or cause-specific death classification (**Figure 1**).

The validation dataset was obtained from The First Affiliated Hospital of Zhengzhou University, a single Chinese center. Patients diagnosed with PC and hospitalized for the first resection between 2012 and 2020 were included in this study. Patients with inaccessible follow-up information were excluded (**Figure 1**).

### Variables

The variables utilized for analysis were age at diagnosis, sex, race record, marital status at diagnosis, tumor size, surgery information, stage, laterality, lymph node (LN) status, and radiotherapy data. Moreover, laboratory and clinicopathologic variables were obtained from our hospital, such as blood calcium, PTH levels, tumor volume, and Ki-67. The TNM staging of PC was not included in this analysis because they were not proposed until 2017 at the eighth edition guideline of AJCC (15).

**Figure 2:**
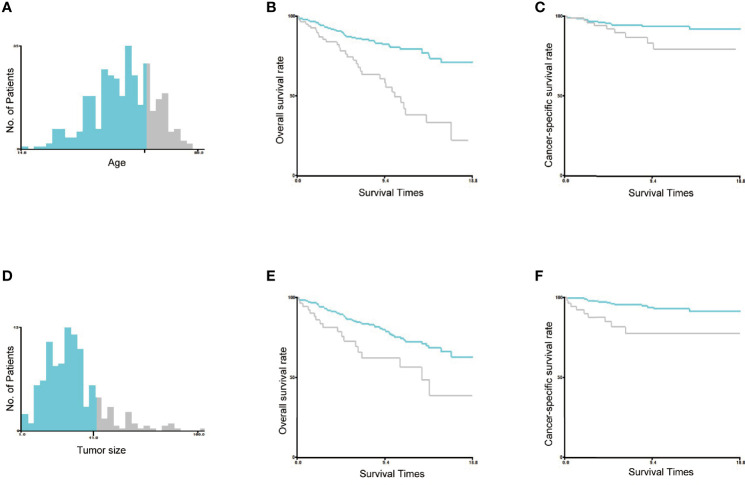
Identification of the optimal cutoff values of age and tumor size. The optimal cutoff values of age were identified as 66 years based on both overall survival and cancer-specific survival **(A–C)**. The optimal cutoff value of tumor size was identified as 41 mm based on both overall survival and cancer-specific survival **(D–F)**.

1) The tumor size included “Extent of Disease (EOD) 10-size (1988–2003),” “Collaborative Stage (CS) tumor size (2004–2015),” and “Tumor Size Summary (2016+)” in the SEER*Stat Software.

2) Age and tumor size were converted into two groups in the training set, and the optimal cutoff was calculated by X-tile software (Version 3.6.1; Robert, MD) (16). As shown in **Figure 2**, the optimal cutoff for age was 66 years, and the optimal cutoff for tumor size was 41 mm. Age was classified as greater than 66 years and less than or equal to 66 years, and tumor size was classified as greater than 41 mm and less than or equal to 41 mm.

3) “Marital status” contained “Married,” “Single,” and “Others” groups. Furthermore, patients in the “Others” group were “Divorced,” “Separated,” Unmarried or Domestic Partner,” or “Widowed,”.

4) The stage was composed of “COMBINED SUMMARY STAGE (2004+)” and “historic stage A (1973–2015).” Furthermore, the staging was aligned to the Summary Stage 2018 Coding Manual v2.0 from the SEER website (**Supplementary Material**) (17). Tumors confined to the parathyroid glands without distant metastasis are called localized group; tumors infiltrating the thyroid, surrounding muscles, recurrent laryngeal nerve, trachea, esophagus, or other tissues and organs without distant metastasis are called regional group; tumors transferring to other organs or distant lymph node were defined as distant metastasis group.

5) Radical surgery referred to the resection extension of the lesion parathyroid, ipsilateral thyroid, and central neck dissection. “Excisional biopsy”, “Local tumor excision, nos (with pathology specimen),” “Photodynamic therapy (PDT),” “Simple/partial surgical removal of primary site,” “debulking,” “Surgery, NOS,” and “Total surgical removal of primary site” were defined as “others.” “None; no cancer-directed surgery of primary site” was defined as “No surgery.”

6) OS and CSS were selected as the outcomes. OS refers to death due to any cause, while CSS represents PC-specific death. The survival months were defined as from the surgery date to November 2020 and June 30, 2021, respectively, for the SEER and Chinese single-center datasets.

### Statistical Analysis

The number and the percentage [N (%)] were used to describe the categorical data, and these data were compared by the chi-square test and Kruskal-Wallis test. The mean ± standard deviation (SD) and median with range were used to describe the normally and non-normally distributed quantitative variable, respectively. Kruskal–Wallis test was used to compare the levels of quantitative variables in different groups. The Kaplan–Meier method was used to estimate the survival and was compared by a log-rank test. The prognostic factors significantly affecting OS and CSS were explored by univariate and multivariate Cox regression analysis. Additionally, the hazard ratio (HR) and 95% confidence interval were reported for prognostic factors, and the receiver operating characteristic (ROC) curves and calibration curves were calculated to validate the nomogram. The bilateral *p* < 0.05 was regarded as significant. All data were analyzed by R software (Version 4.0.3) and IBM SPSS software (Version 22.0).

## Results

### Demographic and Clinical Characteristics

A total of 342 patients from the SEER database and 59 patients from our hospital were identified in this study. The baseline demographics and clinical characteristics are listed in **Table 1**. The mean age of the patients was 55.7 ± 13.8 years, and the mean tumor size was 29.6 ± 15.4 mm. In the training set (n = 342), there were 178 (52%) female patients and 255 (74.6%) white patients; 5 (1.5%) patients did not undergo surgery, and 30 (8.8%) underwent radical surgery treatment. In terms of stage, 18 patients (5.3%) developed distant metastasis. In addition, 112 patients (32.7%) underwent cervical lymph node resection, of whom 10 patients had lymph node metastasis. Moreover, 48 patients (14.0%) received radiation therapy. In the validation set (n = 59), there were 27 men and 32 women, the mean age was 49.0 ± 15.4 years, and the mean tumor size was 32.1 ± 12.7 mm; all populations were Asian; 2 patients’ tumors were “intraglandular parathyroid cancer.” There were no statistically significant differences in gender, age, clinical stage, radiotherapy, overall deaths, and cancer-specific deaths between the training and validation sets (*p* > 0.05). There were differences in marital status (*p* < 0.001), surgery (*p* < 0.001), and lymph node status (*p* = 0.002).

**Table 1:** Demographic and clinical characteristics in the SEER database and a single Chinese center*.

	SEER data	Single-center data	*p* value
	(N = 342)	(N = 59)	
**Sex**			
Women	164 (48.0%)	32 (54.2%)	0.400
Men	178 (52.0%)	27 (45.8%)	
**Age (years)**			
≤66	258 (75.4%)	50 (84.7%)	0.118
>66	84 (24.6%)	9 (15.3%)	
**Race**			
White	255 (74.6%)	NA	NA
Black	53 (15.5%)	NA	
Others^a^	34 (9.9%)	59 (100%)	
**Marital Status**			
Married	219 (64.0%)	56 (94.9%)	<0.001
Single	64 (18.7%)	3 (5.1%)	
Others^b^	59 (17.3%)	NA	
**Tumor Size (mm)**			
1–41	288 (84.2%)	45 (76.3%)	0.136
>41	54 (15.8%)	14 (23.7%)	
**Surgery**			
No surgery	5 (1.5%)	NA	<0.001
Radical surgery	30 (8.8%)	31 (52.5%)	
Others^c^	307 (89.8%)	28 (47.5%)	
**Stage**			
Localized	210 (61.4%)	35 (59.3%)	0.069
Regional	114 (33.3%)	16 (27.1%)	
Distant	18 (5.3%)	8 (13.6%)	
**Laterality**			
Left	23 (6.7%)	29 (49.2%)	<0.001
Right	29 (8.5%)	28 (47.5%)	
Unknown^d^	290 (84.8%)	2 (3.4%)	
**LN Status**			
Negative	102 (29.8%)	31 (52.5%)	0.002
Positive	10 (2.9%)	2 (3.4%)	
Unknown^e^	230 (67.3%)	26 (44.1%)	
**Radiation**			
No	294 (86.0%)	56 (94.9%)	0.058
Yes	48 (14.0%)	3 (5.1%)	
**CSS**			
Alive	319(93.3%)	55 (93.2%)	1
Death	23 (6.7%)	4 (6.8%)	
**OS**			
Alive	267 (78.1%)	49 (83.1%)	0.491
Death	75 (21.9%)	10 (16.9%)	
**Survival months**			
Median [Min, Max]	81.5 [1.0, 226.0]	44.0 [5.0, 117.0]	<0.001

^a^ Including Asian and American Indian

^b^ Including divorced, separated, unmarried, domestic partner, and widowed.

^c^ Including local tumor excision, simple/partial surgical removal of primary site, debulking, etc.

^d^ Without record.

^e^ Regional neck lymphadenectomy not performed.

*SEER, Surveillance, Epidemiology, and End Results; LN, lymph node; CSS, cancer-specific survival; OS, overall survival.

### Prognostic Factors of Overall Survival and Cancer-Specific Survival

As shown in **Table 2**, 10 variables were enrolled for univariate Cox regression analysis. The significant variables were analyzed by multivariate Cox regression (backward methods) later. As shown in **Table 3**, age, marital status, tumor size, stage, LN status, and radiation were identified as independent variables for OS. However, only tumor size and stage were independent factors for CSS.

**Table 2:** Univariate Cox regression analysis of OS and CSS in patients with parathyroid cancer in the training set.

	OS			CSS		
	HR	95% CI	*P*	HR	95% CI	*p*
**Sex**						
Men	**Reference**			**Reference**		
Women	1.580	0.992–2.515	0.054	1.280	0.561–2.922	0.555
**Age**						
≤66	**Reference**			**Reference**		
>66	3.067	1.935–4.863	<0.001	2.414	1.042–5.596	0.040
**Race**						
White	**Reference**			**Reference**		
Black	1.263	0.687–2.324	0.452	1.579	0.574–4.346	0.377
Others^a^	1.743	0.907–3.346	0.095	1.623	0.470–5.608	0.444
**Marital status**						
Married	**Reference**			**Reference**		
Single	1.342	0.713–2.526	0.363	1.540	0.542–4.378	0.418
Others^b^	2.599	1.563–4.323	<0.001	1.904	0.714–5.074	0.198
**Tumor size**						
1–41 mm	**Reference**			**Reference**		
>41 mm	2.177	1.280–3.703	0.004	4.230	1.827–9.790	<0.001
**Surgery**						
No surgery	**Reference**			**Reference**		
Radical surgery	0.169	0.042–0.679	0.012	0.185	0.017–2.057	0.170
Others^c^	0.191	0.060–0.613	0.005	0.187	0.025–1.404	0.103
**Stage**						
Localized	**Reference**			**Reference**		
Regional	1.599	0.973–2.626	0.064	2.173	0.837–5.639	0.111
Distant	5.737	2.889–11.393	<0.001	11.835	4.066–34.455	<0.001
**Laterality**						
Left	**Reference**			**Reference**		
Right	1.211	0.289–5.070	0.794	1.172	0.196–7.021	0.862
Unknown^d^	1.293	0.405–4.126	0.664	0.580	0.134–2.516	0.467
**LN Status**						
Negative	**Reference**			**Reference**		
Positive	6.681	2.465–18.108	<0.001	7.117	1.423–35.785	0.017
Unknown^e^	1.095	0.646–1.855	0.736	1.049	0.407–2.707	0.920
**Radiation**				7.117	1.423–35.785	0.017
No	**Reference**			**Reference**		
Yes	1.814	1.026–3.202	0.041	1.836	0.680–4.958	0.230

^a^ Including Asian and American Indian.

^b^ Including divorced, separated, unmarried, domestic partner, and widowed.

^c^ Including local tumor excision, simple/partial surgical removal of primary site, debulking, etc.

^d^ Without record.

^e^ Regional neck lymphadenectomy not performed.

*OS, overall survival ; CSS, cancer-specific survival; HR, hazard ratio; CI, confidence interval; LN, lymph node.

**Table 3:** Multivariate Cox regression analysis of OS and CSS in patients with parathyroid cancer in the training set.

	OS			CSS		
	HR	95% CI	*p*	HR	95% CI	*p*
**Age**						
≤66	**Reference**					
>66	3.262	1.870–5.689	<0.001			
**Marital status**						
Married	**Reference**					
Single	2.134	1.081–4.212	0.029			
Others^a^	1.745	0.991–3.073	0.054			
**Tumor size**						
1–41 mm	**Reference**			**Reference**		
>41 mm	2.180	1.248–3.810	0.006	3.657	1.566–8.543	0.003
**Marital status**						
Married	**Reference**			**Reference**		
Single	1.160	0.683–1.971	0.583	2.114	0.813–5.494	0.124
Others^b^	3.262	1.870–5.689	<0.001	10.090	3.438–29.614	<0.001
**LN Status**						
Negative	**Reference**					
Positive	6.678	2.318–19.240	<0.001			
Unknown^c^	0.995	0.580–1.706	0.985			
**Radiation**						
No	**Reference**					
Yes	2.075	1.108–3.886	0.023			

^a^ Including divorced, separated, unmarried, domestic partner, and widowed.

^b^Including local tumor excision, simple/partial surgical removal of primary site, debulking, etc.

^c^ Regional neck lymphadenectomy not performed.

*OS, overall survival ; CSS, cancer-specific survival; HR, hazard ratio; CI, confidence interval; LN, lymph node.

### Construction of Nomograms

**Figure 3:**
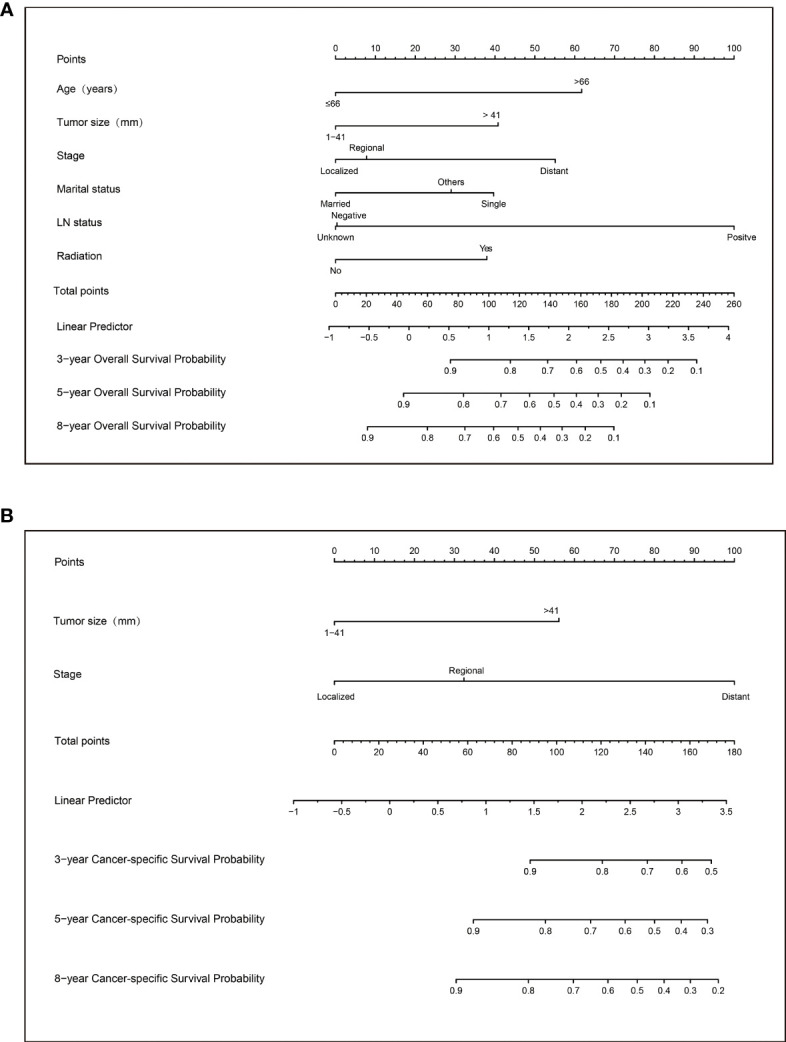
Nomograms to predict 3-, 5-, and 8-year overall survival **(A)** and cancer-specific survival **(B)** for patients with parathyroid carcinoma.

Based on the above variables, we constructed a visualized model to predict the OS and CSS of PC patients at 3, 5, and 8 years. The total score predicts the OS/CSS probabilities through the nomograms and is obtained by adding the score of each variable (**Figure 3**).

### Evaluation and External Validation of Nomograms

**Figure 4:**
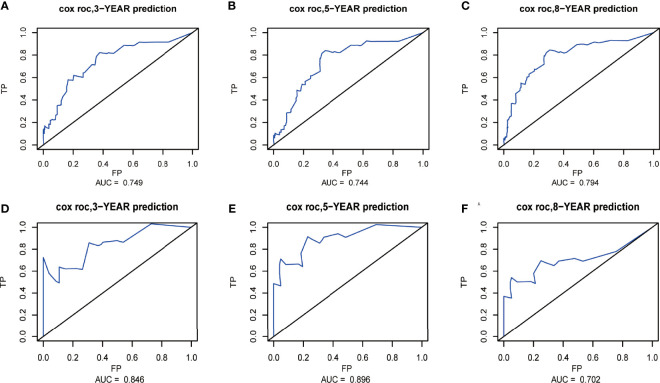
Receiver operating characteristic (ROC) curves at 3, 5, and 8 years in the training set **(A–C)** and validation set **(D–F)** for validating the overall survival (OS) prediction nomogram.

**Figure 5:**
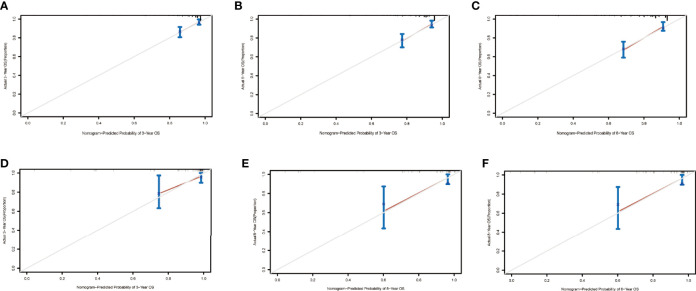
Calibration curves at 3, 5, and 8 years in the training set for validating the overall survival (OS) prediction nomogram **(A–C)**. Calibration curves at 3 and 5 years in the validation set for the OS prediction nomogram **(D–F)**.

**Figure 6:**
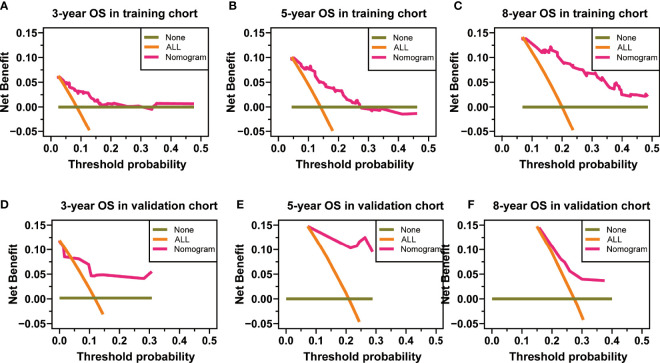
Decision curve analysis at 3, 5, and 8 years in the training set for the overall survival (OS) prediction nomogram **(A–C)**. Decision curve analysis at 3, 5, and 8 years in the validation set for the OS prediction nomogram** (D–F)**.

The nomograms from the SEER set (training set) and Chinese single-center set (validation set) were evaluated and validated by the concordance index (C-index), the area under the curve (AUC), calibration curves, and decision curve analysis (DCA). In the training set, the C-index of the nomogram for OS was 0.736 (95% CI, 0.674–0.798; *p* < 0.001) and 0.820 (95% CI, 0.668–0.972; *p* < 0.001) in the validation set. The AUC of ROC curves in predicting OS at 3, 5, and 8 years in the training set was 0.749, 0.744, and 0.794 **(** **Figures 4A-C** **)**, respectively, while the AUC of ROC curves for the validation set was 0.846, 0.896, and 0.702, respectively (**Figures 4D-F**). The 3-, 5-, and 8-year calibration curves in the training set and validation set for OS prediction proved to have a satisfying fit (**Figure 5**). The ordinate represents the net benefit, and the abscissa is the threshold probability in DCA curves. “All” refers to all interventions, “None” refers to no intervention, and “Nomogram” refers to the intervention under a certain threshold probability. As shown in **Figure 6**, the DCA curves show a better net clinical benefit.

**Figure 7:**
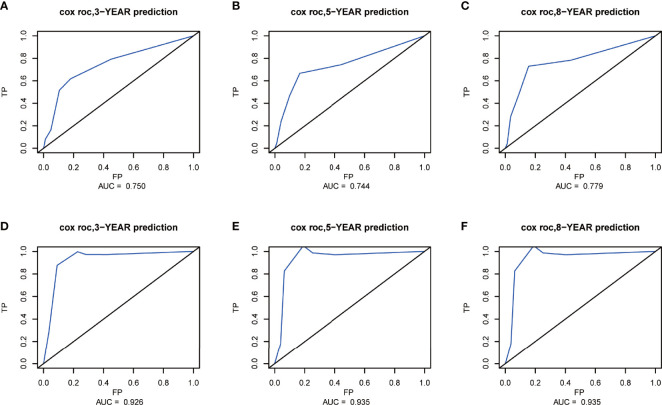
Receiver operating characteristic (ROC) curves at 3, 5, and 8 years in the training set **(A–C)** and validation set **(D–F)** for validating the cancer-specific survival (CSS) prediction nomogram.

**Figure 8:**
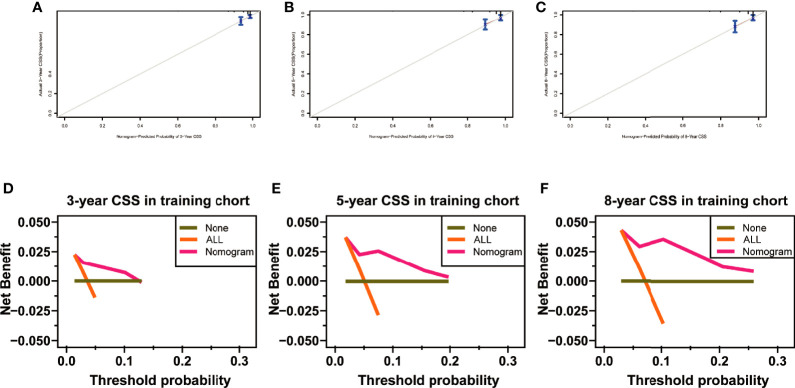
Calibration curves at 3, 5, and 8 years in the training set for validating the cancer-specific survival (CSS) prediction nomogram **(A–C)**. Decision curve analysis at 3, 5, and 8 years in the training set for the CSS prediction nomogram **(D–F)**.

The C-index of the nomogram for CSS was 0.772 (95% CI, 0.661–0.883; *p* < 0.001) in the training set and 0.943 (95% CI, 0.881–1; *p* = 0.003) in the validation set. The AUC of ROC curves in predicting CSS at 3, 5, and 8 years in the training set was 0.750, 0.744, and 0.779, respectively **(** **Figures 7A-C** **)**. The AUC of ROC curves in predicting CSS at 3, 5, and 8 years in the validation set was 0.926, 0.935, and 0.935 (**Figures 7D-F**). Furthermore, the 3-, 5-, and 8-year calibration curves and DCA curves in the training set for CSS prediction proved to have a satisfying fit and net benefit (**Figures 8A-F**). The 3-, 5-, and 8-year CSS calibration curves and DCA curves in the validation set could not be obtained because there were few cancer-specific deaths.

### Web-Based Probability Calculator

**Figure 9:**
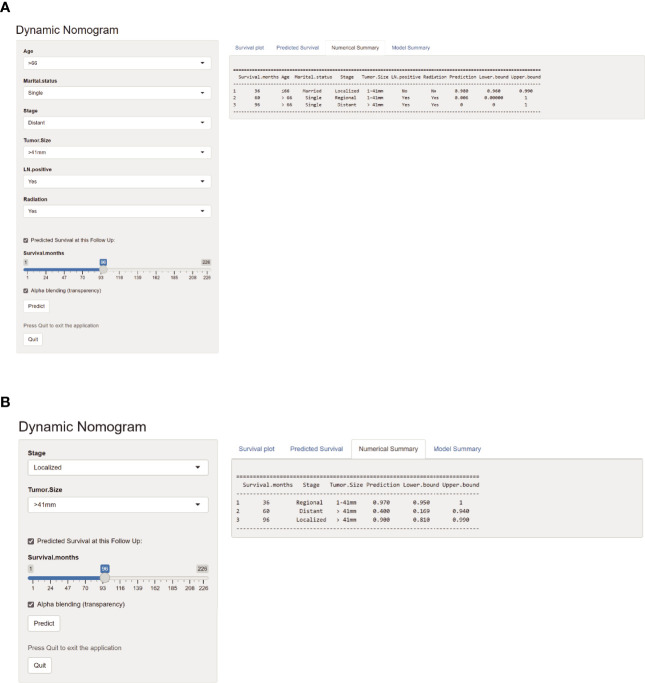
Web-based probability calculators. The 3-, 5-, and 8-year overall survival rate probability was calculated **(A)**. The 3-, 5-, and 8-year cancer-specific survival rate probability was calculated **(B)**.

Applying the above results, dynamic Web-based probability calculators for predicting OS (https://parathyroidcancer.shinyapps.io/dynnomapp/) and CSS (https://parathyroidcancercss.shinyapps.io/dynnomapp/) were constructed (**Figure 3**). The calculators provide a convenient way to predict the individual OS and CSS rates at 3, 5, and 8 years based on PC patient clinical characteristics (**Figure 9**).

### Laboratory and Pathological Indices of Parathyroid Carcinoma Patients

Considering the effects of the stage on OS and CSS of PC, further exploration of the validation set would provide valuable insight. The laboratory and pathologic characteristics of PC patients in our hospital were collected, as shown in **Table 4**. Some variables had missing values, such as correction serum calcium (crCa), albumin (ALB), creatinine (Cr), alkaline phosphatase (ALP), and Ki-67. The postoperative serum calcium and PTH levels were continuously monitored. Patients with distant metastasis was identified in eight cases during the follow-up interval. The detailed information is described in the **Supplementary Material**.

**Table 4:** Laboratory and clinicopathologic indices of parathyroid carcinoma patients in the single Chinese centera.

	Overall	Distant	Regional	Localized
	(N = 59)	(N = 8)	(N = 16)	(N = 35)
**Ca (mmol/L)**				
Mean (SD)	3.36 (0.69)	3.70 (0.77)	3.31 (0.47)	3.31 (0.76)
**crCa (mmol/L)**				
Mean (SD)	3.34 (0.74)	3.85 (0.75)	3.30 (0.46)	3.25 (0.81)
Missing	5 (8.5%)	1 (12.5%)	2 (12.5%)	2 (5.7%)
**P (mmol/L)**				
Median [Min, Max]	0.80 [0.27, 1.91]	1.00 [0.56, 1.48]	0.82 [0.27, 1.04]	0.77 [0.40, 1.91]
**PTH (ng/L)**				
Median [Min, Max]	684 [58.3, 2,690]	1,590 [177, 2,420]	831 [58.3, 2,620]	463 [88.3, 2,690]
**ALB (g/L)**				
Median [Min, Max]	41.2 [34.0, 54.3]	37.4 [34.9, 41.9]	44.8 [34.3, 48.6]	41.3 [34.0, 54.3]
Missing	5 (8.5%)	1 (12.5%)	2 (12.5%)	2 (5.7%)
**Cr (IU/L)**				
Median [Min, Max]	71.4 [6.00, 342]	100 [6.00, 255]	76.5 [28.0, 153]	68.5 [19.0, 342]
Missing	1 (1.7%)	0 (0%)	0 (0%)	1 (2.9%)
**ALP (IU/L)**				
Median [Min, Max]	189 [42.0, 3,590]	233 [64.0, 1,080]	291 [64.0, 3,590]	185 [42.0, 3,320]
Missing	3 (5.1%)	0 (0%)	1 (6.3%)	2 (5.7%)
**Postoperative PTH (ng/L)**				
Median [Min, Max]	18.1 [1.83, 736]	69.1 [9.81, 736]	12.4 [3.62, 58.7]	16.2 [1.83, 199]
**Postoperative Ca (mmol/L)**				
Mean (SD)	2.22 (0.22)	2.18 (0.16)	2.25 (0.22)	2.21 (0.24)
**Tumor Size (mm)**				
Mean (SD)	32.1 (12.7)	42.3 (12.6)	38.2 (12.3)	26.9 (10.4)
**Tumor Volume (mm3)**				
Median [Min, Max]	7,500 [240, 60,000]	12,900 [7,500, 60,000]	16,800 [1,950, 60,000]	4,880 [240, 39,000]
**Ki-67**				
Median [Min, Max]	5 [1, 50]	5 [2, 20]	5 [1, 50]	5 [1, 40]
Missing	11 (18.6%)	1 (12.5%)	2 (12.5%)	8 (22.9%)

^a^ Ca, calcium; crCa, correction serum calcium; P, phosphorus; PTH, parathyroid hormone; ALB, albumin; Cr, creatinine; ALP, alkaline phosphatase.

**Figure 10:**
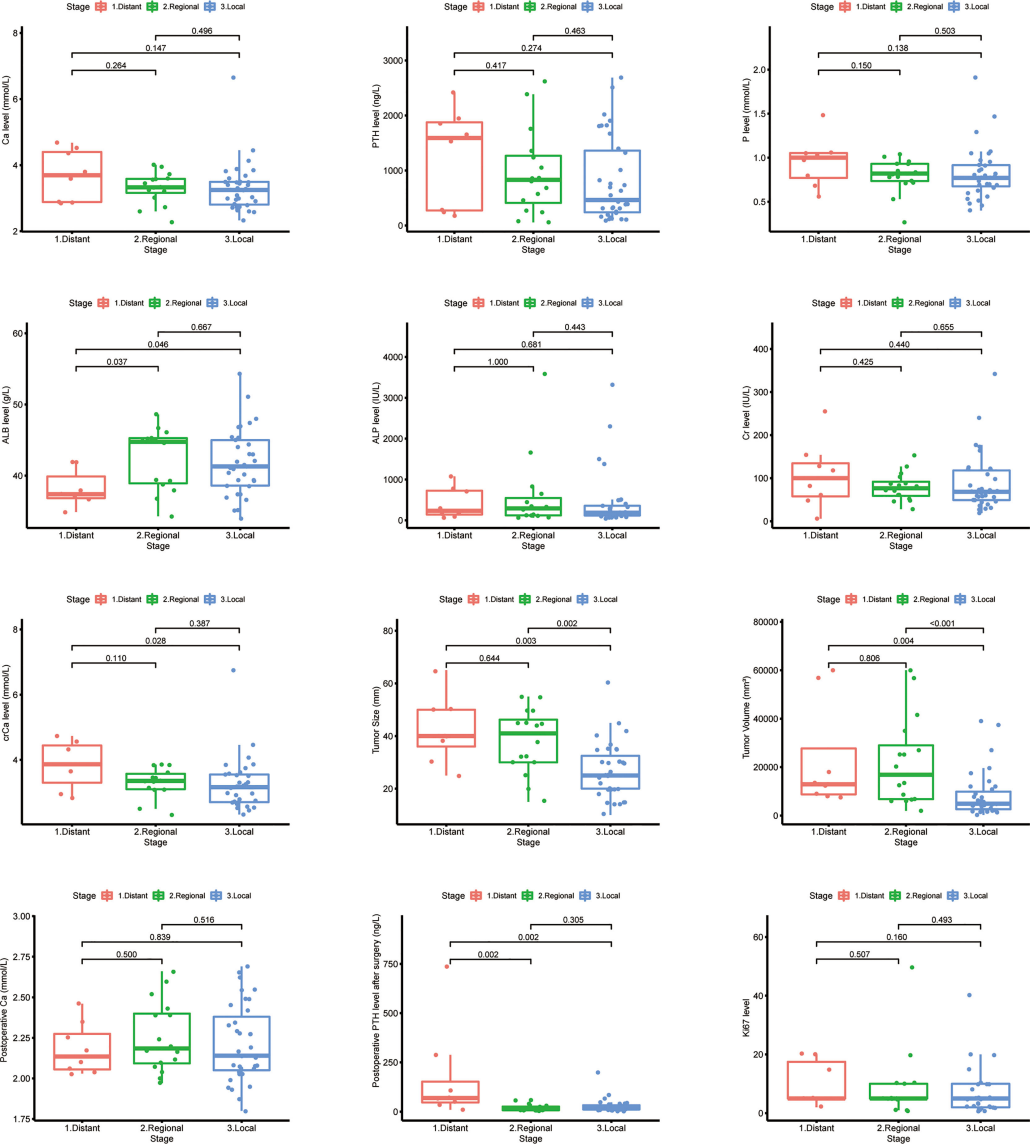
Laboratory and pathologic indicator levels among different stages in Chinese single-center parathyroid carcinoma patients.

The differences between laboratory indicators and Ki-67 between different stages were evaluated (**Figure 10**). The levels of ALB, crCa, tumor size, tumor volume, and postoperative PTH demonstrated statistically significant differences between stages. Lower ALB levels and higher postoperative PTH levels were found in patients with distant metastasis. The tumor size and tumor volume in the local disease group were smaller than those in the regional and distant metastasis groups. The level of crCa in the distant metastasis group was statistically higher than that in the local disease group.

## Discussion

To our knowledge, few studies investigated prognostic factors for patients with PCs, but there was no research that developed a predictive nomogram (11–13, 18–20). This study includes the largest number of patients with a confirmed diagnosis of PC who underwent their first surgery in a single Chinese center. We constructed the nomograms to predict the OS and CSS according to the SEER database, and validation was performed with the Chinese single-center dataset. Extra analysis of the laboratory investigations at different stages was performed. Notably, the prognostic factors for disease-free survival of PC patients in our hospital were reported (21).

There were some differences between the SEER dataset and the Chinese single-center dataset. Some potential causal factors include religious beliefs, economic conditions, incidence, treatment strategy, and so on. Univariate and multivariate Cox regression proportional analysis showed that the independent prognostic factors for OS in patients with PC were age, marital status, tumor size, stage, positive lymph nodes, and radiation therapy. Larger tumor size and distant metastasis were identified as significantly poor prognostic factors of poor CSS.

Age was expected to have an essential role in predicting OS. Additionally, the view that the prognosis of cancer patients is worse with aging is acceptable. Sadler et al. (20) and Silva-Figueroa et al. (12) showed the same effect of age on patients with PC as we did. Older patients tend to have worse comprehensive physical conditions, which results in a higher incidence of complications after surgery and a longer recovery time (22, 23). Therefore, better general geriatric medical management could lead to improved OS of PC patients.

Our finding indicates that marital status was associated with the OS of PC patients, and married patients had a better prognosis than that of single or other patients. Marital status is not only an independent prognostic indicator of many cancers (24–27) but also a risk factor for developing cancers (28, 29). A variety of reasons may lead to this result. With family support or spouse support, married patients were better equipped to overcome cancer better than patients with other marital statuses. Having relatively strong financial resources makes it easier to obtain better treatment for married patients, which is associated with a better prognosis. Our findings suggest that married PC patients have a better prognosis than other patients. However, we have no information on socioeconomic status available for prediction. It highlights the potentially significant impact that social support can have on survival. Nevertheless, these results still needed to be verified.

PC is a radioresistant tumor, and there is no indication that radiotherapy is performed as a regular treatment (30). The PC analysis in the National Cancer Data Base (NCDB) showed that radiotherapy did not improve the OS rate of patients (31). Among the largest series of patients published, the 5-year OS rate of patients receiving radiotherapy is lower. In contrast, this may reflect those patients with the more advanced disease who are referred for radiotherapy (20). The same result was observed in this study. Christakis et al. (32) showed that long-term disease control for high-risk patients could be achieved through reoperation and postoperative radiotherapy at Anderson Cancer Center. The effect of radiotherapy should be investigated in a long-term study including more patients, as there are only 8 cases in the above study (32).

It is controversial that a positive lymph node status is associated with a poor prognosis. However, this study and others revealed that lymph nodes status is significantly associated with poor prognosis (20, 33–35). Less than a third of the patients (**Table 1**) undergoing neck dissection have had the lymph nodes investigated. Therefore, a considerable proportion of patients likely have insufficient staging of the lymphatic involvement of the disease.

Tumor size and stage have always been considered the key factors affecting the prognosis of PC (3, 13, 18). The most common cutoff value of tumor size was recognized as 3 cm, although it was 4.1 cm in this study (10). This may be related to the number of patients and outcome indicators in different studies. The involvement of distant organs or lymph nodes in parathyroid cancer indicates poor outcomes. The OS and CSS Kaplan–Meier curves of the distant metastasis group showed a significant difference from the other in the validation set. Mostly, distant metastasis patients without reoperation suffered from hypercalcemia (36).

Therefore, the early identification of distant metastasis is valuable in parathyroid cancer management. Patients with distant metastases were likely to have lower ALB levels and higher postoperative PTH levels compared to other patients. The correlation between serum ALB and tumor prognosis was found in various tumors (37). A meta-analysis showed that lower ALB levels before treatment were significantly associated with poorer metastasis-free survival in nasopharyngeal carcinoma (38). In addition, lower ALB levels in gastric, rectal, and cervical cancers have also been shown to be prognostic predictors of tumors (39–41). ALB is closely associated with inflammatory and dystrophic status in cancer patients. ALB is mainly synthesized by the liver and is the most and smallest molecular in plasma (42). They also activate DNA replication in cancer cells, thereby promoting tumor proliferation and immune escape (43, 44). Thus, PC can lead to lower ALB levels by inducing malnutrition and anorexia, further leading to disease progression and forming a vicious circle. When evaluating PC patients, it is recommended that serum ALB levels be routinely checked and adequately evaluated accordingly. And for metastatic parathyroid cancer patients, early nutritional intervention to correct ALB levels may be a new idea.

Several limitations of this study should be addressed. Firstly, since this is a retrospective study, information and selection bias may be present. Secondly, the eighth edition of the AJCC staging system and much clinicopathological information, such as recurrence and calcium and PTH levels, and some individual molecular factors were not found in the SEER database. Lastly, the classification of age and tumor size based on this study may not apply to other studies. Larger cohorts are necessary for further validation of the present nomograms.

In conclusion, predictive nomograms integrating independent prognostic factors and Web-based probability calculators based on the SEER database were constructed to predict the OS and CSS rates of PC patients. The established nomogram application can provide accurate prognostics for patients with PC in the Chinese population, but it must be validated on prospectively collected real-world data.

## Data Availability Statement

The original contributions presented in the study are included in the article/**Supplementary Material**. Further inquiries can be directed to the corresponding author.

## Ethics Statement

This research was approved by the Ethics Committee of the First Affiliated Hospital of Zhengzhou University (Number: 2021-KY-1062-001). Patients from the SEER database had consented to be studied publicly in any scientific research worldwide.

## Author Contributions

MT had full access to all of the data from both SEER and our hospital, performed the statistical analysis, and drafted the article. SL took responsibility for the data integrity and interpretation. XW and MJ verified the accuracy of the data and performed proofreading. XL supervised and proofread. All authors participated in the concept and design. All authors contributed to the article and approved the submitted version.

## Funding

This work was supported by the 2019 Henan Provincial Medical Science and Technology Research Plan Joint Provincial and Ministerial Project (No. SB201901023).

## Conflict of Interest

The research was conducted in the absence of any commercial or financial relationships that could be construed as a potential conflict of interest.

## Publisher’s Note

All claims expressed in this article are solely those of the authors and do not necessarily represent those of their affiliated organizations, or those of the publisher, the editors and the reviewers. Any product that may be evaluated in this article, or claim that may be made by its manufacturer, is not guaranteed or endorsed by the publisher.

## Footnotes

1 Including Asian and American Indian.

2 Including divorced, separated, unmarried, domestic partner, and widowed.

3 c Including local tumor excision, simple/partial surgical removal of primary site, debulking, etc.

4 d Without record.

5 e Regional neck lymphadenectomy not performed.

6 Including Asian and American Indian.

7 Including divorced, separated, unmarried, domestic partner, and widowed.

8 Including local tumor excision, simple/partial surgical removal of primary site, debulking, etc.

9 Including divorced, separated, unmarried, domestic partner, and widowed.

10 Including local tumor excision, simple/partial surgical removal of primary site, debulking, etc.
