# Supplementary material for: A Nomogram Predicting the Overall Survival and Cancer-Specific Survival in Patients with Parathyroid Cancer: A Retrospective Study
Source: Front Endocrinol (Lausanne). 2022 May 19;13:850457. doi: 10.3389/fendo.2022.850457 (PMC9160525; doi:10.3389/fendo.2022.850457)
Supplement: Supplementary file 1 [file DataSheet_1.pdf]

ENDOCRINE SYSTEM

THYROID

8000-8700, 8720-8790, 9700-9701

C739
C739 Thyroid gland

Note 1: The following sources were used in the development of this chapter

- SEER Extent of Disease 1988: Codes and Coding Instructions (3rd Edition, 1998) (https://seer.cancer.gov/archive/manuals/EOD10Dig.3rd.pdf)
- SEER Summary Staging Manual-2000: Codes and Coding Instructions (https://seer.cancer.gov/tools/ssm/ssm2000/)
- Collaborative Stage Data Collection System, version 02.05: https://cancerstaging.org/cstage/Pages/default.aspx
- Chapter 73 *Thyroid-Differentiated and Anaplastic Carcinoma*, in the AJCC Cancer Staging Manual, Eighth Edition (2017) published by Springer International Publishing. Used with permission of the American College of Surgeons, Chicago, Illinois.
- Chapter 74 *Thyroid-Medullary*, in the AJCC Cancer Staging Manual, Eighth Edition (2017) published by Springer International Publishing. Used with permission of the American College of Surgeons, Chicago, Illinois.

Note 2: See the following chapters for the listed histologies

- 8710-8714, 8800-8934, 8940-9138, 9141-9582: *Soft Tissue*
- 8935-8936: *GIST*
- 9140: *Kaposi Sarcoma*

SUMMARY STAGE

0 In situ: noninvasive, intraepithelial

1 Localized only (localized, NOS)

- Confined to thyroid, NOS
- Into thyroid capsule, but not beyond
- Multiple foci confined to thyroid
- Single invasive tumor confined to thyroid

2 Regional by direct extension only

- Blood vessel(s) (major)
  - Carotid artery (encased)
  - Jugular vein
  - Thyroid artery or vein
- Cricoid cartilage
- Esophagus
- Extrathyroidal extension (microscopic, macroscopic, NOS)
- Larynx
- Nerves
  - Recurrent laryngeal
  - Vagus nerve
- Parathyroid
- Pericapsular soft tissue/connective tissue
- Sternocleidomastoid muscle
- Strap muscle(s)
  - Omohyoid
  - Sternohyoid
  - Sternothyroid
  - Thyrohyoid
- Subcutaneous soft tissue
- Thyroid cartilage
- Trachea
- Tumor described as "FIXED to adjacent tissues"

3 Regional lymph node(s) involved only

- Level I
  - Level IA - Submental
  - Level IB - Submandibular (submaxillary), sublingual
- Level II - Upper jugular
  - Jugulodigastric (subdigastric)
  - Upper deep cervical
  - Level IIA - Anterior
  - Level IIB - Posterior
- Level III - Middle jugular
  - Middle deep cervical
- Level IV - Lower jugular
  - Jugulo-omohyoid (supraomohyoid)
  - Lower deep cervical
  - Virchow node
- Level V - Posterior triangle group
  - Posterior cervical
  - Level VA - Spinal accessory

        - Level VB - Transverse cervical, supraclavicular
- Level VI - Anterior compartment group
    - Laterotracheal
    - Paralaryngeal
    - Paratracheal - above suprasternal notch
    - Perithyroidal
    - Precricoid (Delphian)
    - Prelaryngeal
    - Pretracheal - above suprasternal notch
    - Recurrent laryngeal
- Level VII - Superior mediastinal group (for other mediastinal node(s) see code 7)
    - Esophageal groove
    - Paratracheal - below suprasternal notch
    - Pretracheal - below suprasternal notch
- Other groups
    - Cervical, NOS
    - Deep cervical, NOS
    - Facial
        - Buccinator (buccal)
        - Mandibular
        - Nasolabial
    - Internal jugular, NOS
    - Parapharyngeal
    - Parotid
        - Infraauricular
        - Intraparotid
        - Periparotid
        - Preauricular
    - Retroauricular (mastoid)
    - Retropharyngeal
    - Suboccipital
- Regional lymph node(s), NOS
    - Lymph node(s), NOS

4 Regional by BOTH direct extension AND regional lymph node(s) involved

- Codes (2) + (3)

7 Distant site(s)/lymph node(s) involved

- Distant site(s) (including further contiguous extension)
    - Gross extrathyroidal extension invading
        - Bone
        - Mediastinal tissues

        - Prevertebral fascia
        - Skeletal muscle, other than strap or sternocleidomastoid muscle
- Distant lymph node(s), NOS
- Distant metastasis, NOS
    - Carcinomatosis
    - Distant mets WITH or WITHOUT distant lymph node(s)

9 Unknown if extension or metastasis

PARATHYROID

8000-8700, 8720-8790, 9700-9701

C750
C750 Parathyroid

Note 1: The following sources were used in the development of this chapter

- SEER Extent of Disease 1988: Codes and Coding Instructions (3rd Edition, 1998) (https://seer.cancer.gov/archive/manuals/EOD10Dig.3rd.pdf)
- SEER Summary Staging Manual-2000: Codes and Coding Instructions (https://seer.cancer.gov/tools/ssm/ssm2000/)
- Collaborative Stage Data Collection System, version 02.05: https://cancerstaging.org/cstage/Pages/default.aspx
- Chapter 75 *Parathyroid*, in the AJCC Cancer Staging Manual, Eighth Edition (2017) published by Springer International Publishing. Used with permission of the American College of Surgeons, Chicago, Illinois.

Note 2: See the following chapters for the listed histologies

- 8710-8714, 8800-8934, 8940-9138, 9141-9582: *Soft Tissue*
- 8935-8936: *GIST*
- 9140: *Kaposi Sarcoma*

Note 3: Parathyroid tumors are defined as left or right and superior (upper) or inferior (lower).

Note 4: Atypical parathyroid neoplasms (code 0) are defined as tumors that are histologically or clinically worrisome but do not fulfill the more robust criteria [i.e., invasion metastasis] for carcinoma.

Note 5: Metastases for the parathyroid is anything beyond the central and lateral part of the neck.

SUMMARY STAGE

0 In situ, intraepithelial, noninvasive

- Atypical parathyroid neoplasm (neoplasm of uncertain malignant potential)

1 Localized only (localized, NOS)

- Confined to parathyroid
- Extension to soft tissue

2 Regional by direct extension only

- Adjacent lymph nodes
- Esophagus
- Recurrent laryngeal nerve
- Thymus
- Thyroid gland
- Trachea
- Skeletal muscle

3 Regional lymph node(s) involved only

- Level I
  - Level IA - Submental
  - Level IB - Submandibular (submaxillary), sublingual
- Level II - Upper jugular
  - Jugulodigastric (subdigastric)
  - Upper deep cervical
  - Level IIA - Anterior
  - Level IIB - Posterior
- Level III - Middle jugular
  - Middle deep cervical
- Level IV - Lower jugular
  - Jugulo-omohyoid (supraomohyoid)
  - Lower deep cervical
  - Virchow node
- Level V - Posterior triangle group
  - Posterior cervical
  - Level VA - Spinal accessory
  - Level VB - Transverse cervical, supraclavicular
- Level VI - Anterior compartment group
  - Laterotracheal
  - Paralaryngeal
  - Paratracheal - above suprasternal notch
  - Perithyroidal
  - Precricoid (Delphian)
  - Prelaryngeal
  - Pretracheal - above suprasternal notch
  - Recurrent laryngeal
- Level VII - Superior mediastinal group (for other mediastinal node(s) see code 7)
  - Esophageal groove
  - Paratracheal - below suprasternal notch
  - Pretracheal - below suprasternal notch
- Other groups
  - Cervical, NOS

    - Deep cervical, NOS
    - Facial
        - Buccinator (buccal)
        - Mandibular
        - Nasolabial
    - Internal jugular, NOS
    - Parapharyngeal
    - Parotid
        - Infraauricular
        - Intraparotid
        - Periparotid
        - Preauricular
    - Retroauricular (mastoid)
    - Retropharyngeal
    - Suboccipital
- Regional lymph node(s), NOS
    - Lymph node(s), NOS

4 Regional by BOTH direct extension AND regional lymph node(s) involved

- Codes (2) + (3)

7 Distant site(s)/lymph node(s) involved

- Distant site(s) (including further contiguous extension)
    - Bone
    - Diaphragm
    - Liver
    - Lung
    - Pancreas
    - Spleen
- Distant lymph node(s), NOS
- Distant metastasis, NOS
    - Carcinomatosis
    - Distant metastasis WITH or WITHOUT distant lymph node(s)

9 Unknown if extension or metastasis

ADRENAL GLAND

8000-8700, 8720-8790, 9700-9701

C740-C741, C749, C755
C740 Cortex of adrenal gland
C741 Medulla of adrenal gland
C749 Adrenal gland, NOS
C755 Aortic body and other paraganglioma (*Histologies 8680, 8690, 8692-8693, 8700 only*)

Note 1: The following sources were used in the development of this chapter

- SEER Extent of Disease 1988: Codes and Coding Instructions (3rd Edition, 1998) (https://seer.cancer.gov/archive/manuals/EOD10Dig.3rd.pdf)
- SEER Summary Staging Manual-2000: Codes and Coding Instructions (https://seer.cancer.gov/tools/ssm/ssm2000/)
- Collaborative Stage Data Collection System, version 02.05: https://cancerstaging.org/cstage/Pages/default.aspx
- Chapter 76 *Adrenal Cortical Carcinoma*, in the AJCC Cancer Staging Manual, Eighth Edition (2017) published by Springer International Publishing. Used with permission of the American College of Surgeons, Chicago, Illinois.
- Chapter 77 *Adrenal-Neuroendocrine Tumors*, in the AJCC Cancer Staging Manual, Eighth Edition (2017) published by Springer International Publishing. Used with permission of the American College of Surgeons, Chicago, Illinois.

Note 2: See the following chapters for the listed histologies

- 8710-8714, 8800-8934, 8940-9138, 9141-9582: *Soft Tissue*
- 8935-8936: *GIST*
- 9140: *Kaposi Sarcoma*

SUMMARY STAGE

0 In situ: noninvasive, intraepithelial

1 Localized only (localized, NOS)

- Confined to adrenal gland, no extra-adrenal invasion

2 Regional by direct extension only

- Adjacent connective tissue
- Gerota's fascia
- Invasion of adjacent organs
    - Blood vessels (large)

        - Renal vein
        - Vena cava
    - Kidney
    - Retroperitoneal structures including
        - Great vessels (aorta, inferior vena cava)

3 Regional lymph node(s) involved only

- Aortic, NOS
    - Para-aortic
    - Periaortic
- Pericaval, NOS
    - Paracaval
    - Precaval
    - Retrocaval
- Retroperitoneal, NOS
- Regional lymph node(s), NOS
    - Lymph node(s), NOS

4 Regional by BOTH direct extension AND regional lymph node(s) involved

- Codes (2) + (3)

7 Distant site(s)/lymph node(s) involved

- Distant site(s) (including further contiguous extension)
    - Bone
    - Diaphragm
    - Liver
    - Lung
    - Pancreas
    - Spleen
- Distant lymph node(s), NOS
- Distant metastasis, NOS
    - Carcinomatosis
    - Distant metastasis WITH or WITHOUT distant lymph node(s)

9 Unknown if extension or metastasis

ENDOCRINE OTHER

8000-8700, 8720-8790, 9700-9701 (C754, C758-C759)
8000-8671, 8681-8683, 8691, 8720-8790, 9700-9701 (C755)

C754-C755, C758-C759
C754 Carotid body
C755 Aortic body and other paraganglia
C758 Overlapping lesion of endocrine glands and related structures
C759 Endocrine gland, NOS

Note 1: The following sources were used in the development of this chapter

- SEER Extent of Disease 1988: Codes and Coding Instructions (3rd Edition, 1998) (https://seer.cancer.gov/archive/manuals/EOD10Dig.3rd.pdf)
- SEER Summary Staging Manual-2000: Codes and Coding Instructions (https://seer.cancer.gov/tools/ssm/ssm2000/)
- Collaborative Stage Data Collection System, version 02.05: https://cancerstaging.org/cstage/Pages/default.aspx

Note 2: See the following chapters for the listed histologies

- 8680, 8690, 8692-8693, 8700 (C755 only): *Adrenal Gland*
- 8710-8714, 8800-8934, 8940-9138, 9141-9582: *Soft Tissue*
- 8935-8936: *GIST*
- 9140: *Kaposi Sarcoma*

Note 3: Summary Stage is the only applicable staging system for this site/histology/schema.

SUMMARY STAGE

0 In situ: noninvasive, intraepithelial

1 Localized only (localized, NOS)

- Invasive tumor confined to gland of origin

2 Regional by direct extension only

- Adjacent connective tissue
- Adjacent organs/structures
  - Aortic body
    - Organs/structures in mediastinum
  - Carotid body
    - Upper neck

3 Regional lymph node(s) involved only

- Cervical for carotid body
- Mediastinal for aortic body
- Regional lymph node(s), NOS
  - Lymph node(s), NOS

4 Regional by BOTH direct extension AND regional lymph node(s) involved

- Codes (2) + (3)

7 Distant site(s)/lymph node(s) involved

- Distant site(s) (including further contiguous extension)
- Distant lymph node(s), NOS
- Distant metastasis, NOS
  - Carcinomatosis
  - Distant metastasis WITH or WITHOUT distant lymph node(s)

9 Unknown if extension or metastasis
